# Evaluation of B-Mode and Color Doppler Ultrasound in the Diagnosis of Malignant Cervical Lymphadenopathy

**DOI:** 10.7759/cureus.9819

**Published:** 2020-08-17

**Authors:** Kamat Rohan, Ananthakrishnan Ramesh, Sathasivam Sureshkumar, Chellappa Vijayakumar, KM Abdulbasith, Balamourougan Krishnaraj

**Affiliations:** 1 Radiodiagnosis, Jawaharlal Institute of Postgraduate Medical Education and Research, Puducherry, IND; 2 Surgery, Jawaharlal Institute of Postgraduate Medical Education and Research, Puducherry, IND

**Keywords:** cervical lymph nodes, b mode and colour doppler ultrasound, short-axis diameter (sad), short axis / long-axis diameter ratio (s/l), echogenic hilum, peripheral/mixed vascularity

## Abstract

Background

B-mode ultrasound (BMUS) and color doppler ultrasound (CDUS) could be valuable in evaluating cervical lymphadenopathy compared to palpation. This study aimed at evaluating the efficacy of BMUS and CDUS in differentiating cervical lymph nodes into benign and malignant nature.

Methodology

In this cross-sectional analytical study, a total of 166 patients, who were referred for US-guided fine-needle aspiration cytology (FNAC) of cervical nodes, were included. Patients with cystic/ necrotic cervical nodes or without FNAC/biopsy report were excluded. All study patients underwent BMUS and CDUS, followed by the reference investigation of FNAC/biopsy for analysis. In BMUS, short-axis diameter (SAD), short-axis/long-axis diameter ratio (S/L), presence or absence of echogenic hilum and well defined or ill-defined borders were analyzed. In CDUS, the vascular pattern of a cervical node was categorized as hilar, peripheral or mixed. In cases with multiple cervical lymph nodes, the node having the most suspicious features on the greyscale US was chosen. The results were compared with the final FNAC/biopsy reports.

Results

A total of 166 patients were analyzed in this study. The cut-off point of SAD and S/L ratio for the cervical lymph nodes was 1.28cm and 0.595. The S/L ratio was the best BMUS parameter with a sensitivity of 75%, the specificity of 81%, and an accuracy of 79%. Loss of echogenic hilum was the most sensitive parameter in this study with a sensitivity of 95.4% and an accuracy of 79.5%. The presence of ill-defined margins was significantly higher in the malignant nodes than the benign nodes with a p-value <0.001. The presence of peripheral/mixed vascularity was higher in the malignant nodes than the benign nodes with a p-value <0.001.

Conclusions

Malignant nodes had significantly higher SAD, higher S/L ratio, loss of echogenic hilum, presence of ill-defined margins and peripheral/mixed vascularity compared to benign nodes. The loss of echogenic hilum was the most accurate and sensitive parameter, while the S/L ratio was found to be the most specific BMUS parameter in the detection of malignant nodes. BMUS and CDUS identify malignant nodes and also helps in guiding FNAC/biopsy.

## Introduction

Cervical lymphadenopathy may be due to a large number of causes. A few common causes are tuberculosis, reactive lymphoid hyperplasia, lymphomas and metastasis [1,2]. Radiological differentiation of metastatic nodes from their benign or reactive counterparts is an essential part of clinical workup influencing the treatment and the prognosis [1,3,4].

Grayscale ultrasound (US) gives information about the cervical nodal morphological character like size, shape, borders, echogenicity, etc. [5]. CT and MRI may not be as useful as the US in assessing the internal characteristics of the cervical nodes [5]. Certain radiological features help distinguish the benign and malignant nodes based on B-mode US (BMUS) and color doppler US (CDUS)

Elastography and contrast-enhanced US (CEUS) are advanced non-invasive imaging techniques that can differentiate benign and malignant lymph nodes. Still, they are not readily available in a primary or secondary care setting and need more expertise and training. However, there is a need for an imaging technique that will more reliably and non-invasively differentiate them without radiation risks or significant cost. Hence this study was carried to assess the diagnostic role of BMUS and CDUS in differentiating benign and malignant cervical lymph nodes.

## Materials and methods

This study was a cross-sectional analytical study conducted for two years in a tertiary care center in South India. Institute human ethics committee (IEC) approval was obtained and informed consent was taken before enrolling patients. Patients referred for US-guided fine-needle aspiration cytology (FNAC) for cervical nodes, were included in this study. Patients with cystic/necrotic cervical nodes or without FNAC/biopsy report were excluded. All study patients underwent FNAC after the BMUS and CDUS. When patients have multiple cervical lymph nodes, the node that appears most suspicious on the greyscale US was selected for the sake of the study.

BMUS and CDUS were performed with the Acuson S3000 US system (Siemens, Erlangen, Germany) by an experienced radiologist. A linear array transducer (9L4) with 9MHz frequency was used. The patients were examined in the supine position with the extension of the neck. In a BMUS examination, the parameters assessed were short-axis diameter (SAD), short-axis/long-axis diameter ratio (S/L), presence or absence of echogenic hilum and well defined or ill-defined borders. In CDUS, the vascular pattern of a cervical node was categorized as hilar, peripheral or mixed (showing both hilar and peripheral flow patterns). US-guided FNAC/biopsy was performed in all nodes studied by BMUS and CDUS. In patients where both FNAC and trucut biopsy were done, the reports obtained by the latter were used for analysis. An experienced pathologist who was blinded to the imaging findings reported all the FNAC/biopsies. 

Statistical analysis

The comparison of continuous variables was carried out using Independent Student’s T-test for normally distributed data and Mann-Whitney U test for non-normally distributed data. For comparing the categorical data, the Chi-Square test or Fischer’s exact test was used. Receiver Operator Characteristic (ROC) was plotted to evaluate the diagnostic value of BMUS in detecting malignant lymph nodes. The diagnostic role of BMUS and CDUS was assessed by calculating sensitivity, specificity and predictive values. All the statistical tests were performed on Statistical Package for the Social Sciences (SPSS) software (version 19.0) (IBM Corp., Armonk, NY) and a p-value of less than 0.05 was deemed statistically significant.

## Results

A total of 166 patients were included in the study based on the inclusion and exclusion criteria. The male to female ratio was not comparable in study patients (95 vs. 71). The mean age of study patients was 42.51 ± 18.88 years. Benign and malignant nodes were equally [52% (n=87) vs. 48% (n=79)] distributed in the final HPE reports (Table 1).

**Table 1:** Results of FNAC/biopsy from the cervical lymph nodes FNAC: fine-needle aspiration cytology

Etiopathological diagnosis (N=166)	Frequency [N (%)]
Reactive lymph nodes	53 (32%)
Metastatic squamous cell carcinomas	35 (21%)
Tuberculous/granulomatous nodes	24 (14.4%)
Metastatic adenocarcinomas	17 (10.2%)
Hodgkin and Non-Hodgkin lymphomas	17 (10.2%)
Metastatic thyroid carcinomas	7(4.2%)
Metastatic undifferentiated carcinomas	5 (3%)
Chronic lymphoid leukaemia	2 (1.2%)
Others	6 (3.6%)

BMUS and CDUS

**Figure 1:**
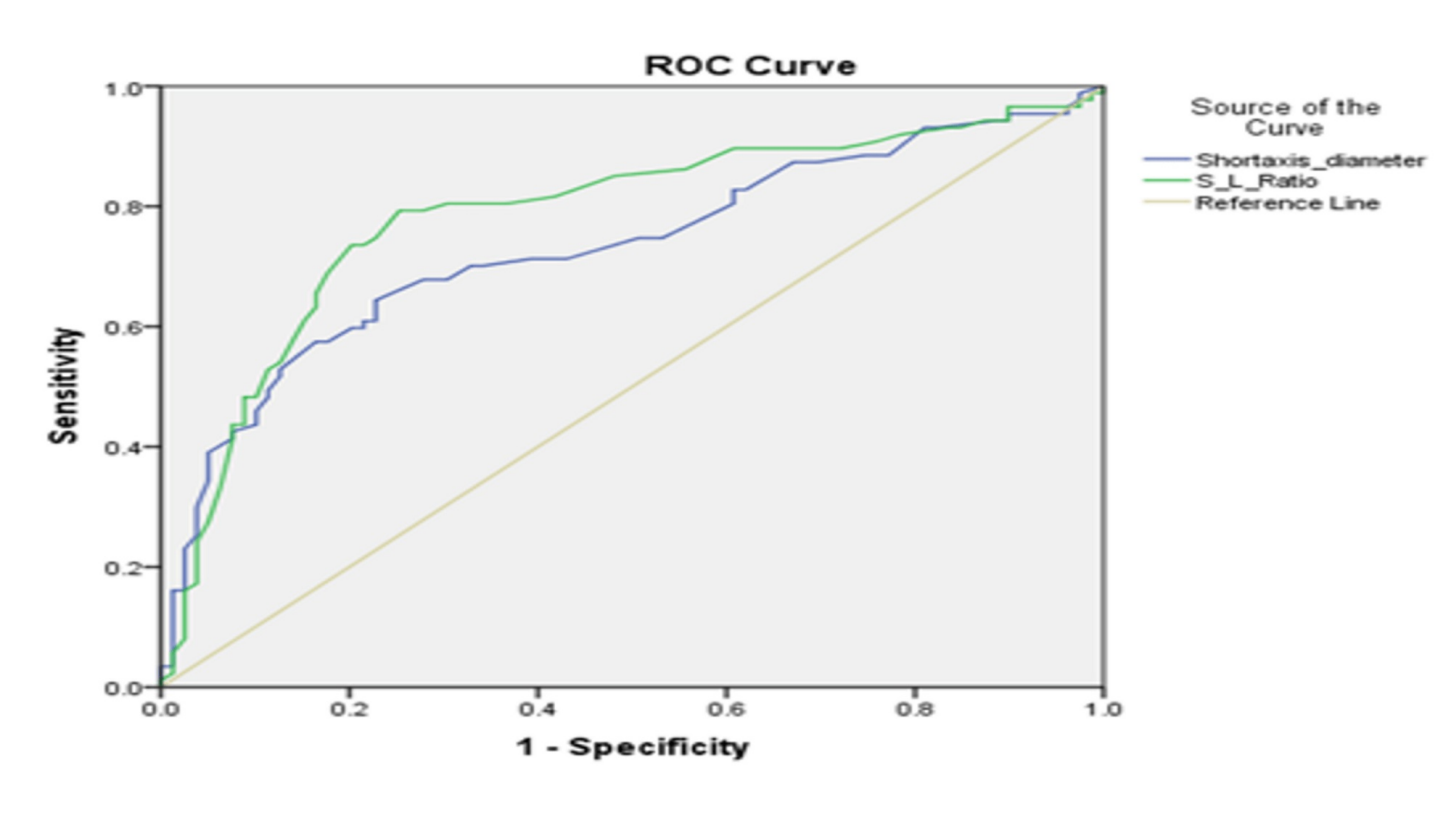
ROC curve of SAD and S/L ratio ROC: receiver operator characteristic; SAD: short-axis diameter; S/L: short-axis to long-axis ratio

The cut-off point of SAD and S/L ratio for the cervical lymph nodes was 1.28cm and 0.595, respectively, which were calculated from the ROC curves (Figure 1).

**Figure 2:**
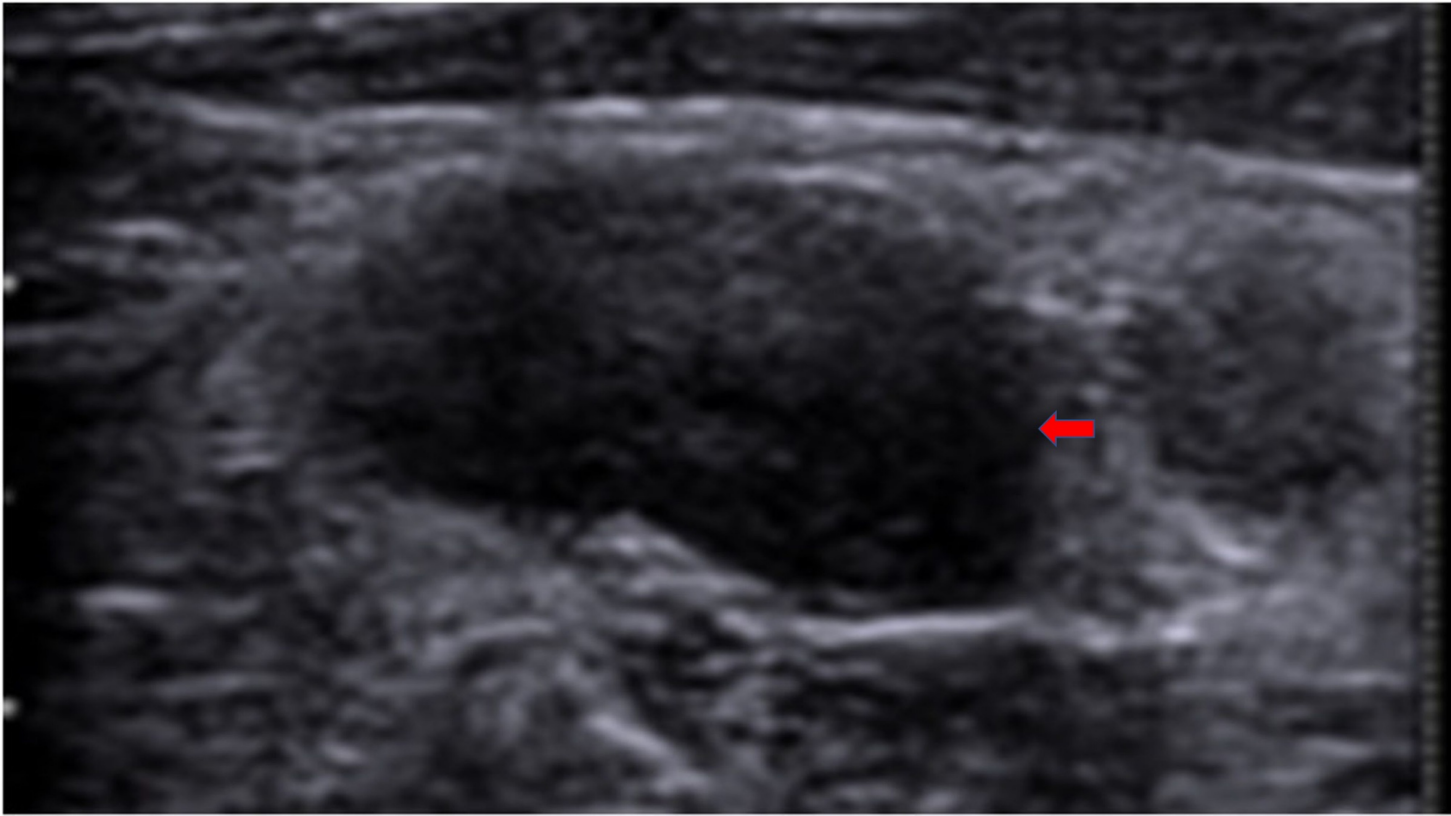
Reactive cervical lymph node shows an oval node with well-defined borders and S/L ratio of <0.5 (red arrow) S/L: short-axis to long-axis ratio

In this study, reactive and benign lymph nodes were oval-shaped, and hence their S/L ratio was <0.5 (Figure 2).

**Figure 3:**
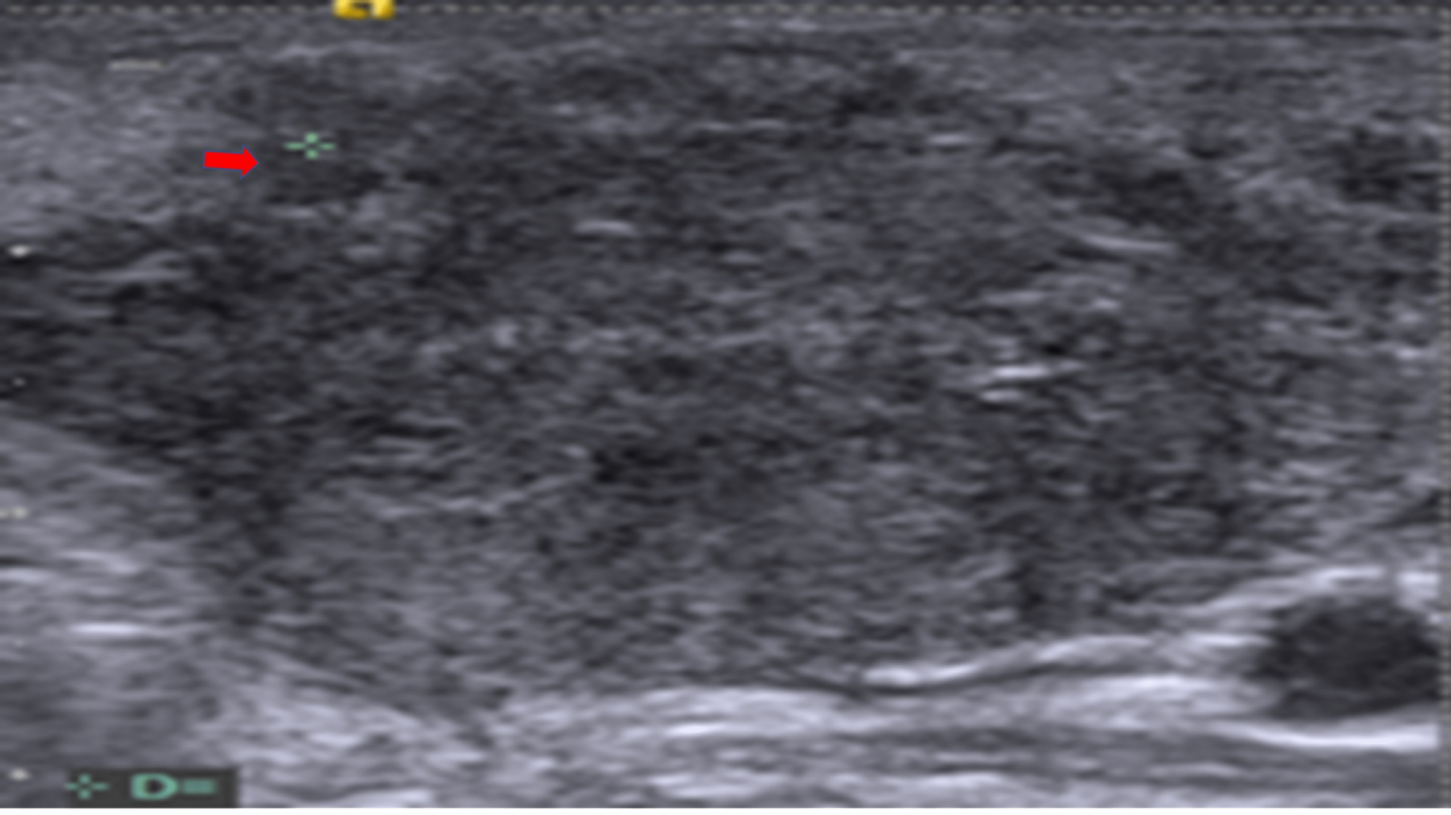
Malignant cervical node shows irregular/ill-defined borders more or less round shape with S/L ratio of >0.5 (red arrow) S/L: short-axis to long-axis ratio

Malignant lymph nodes of study patients were rounded, with an L/S ratio of <2 or an S/L ratio >0.5 (Figure 3).

**Figure 4:**
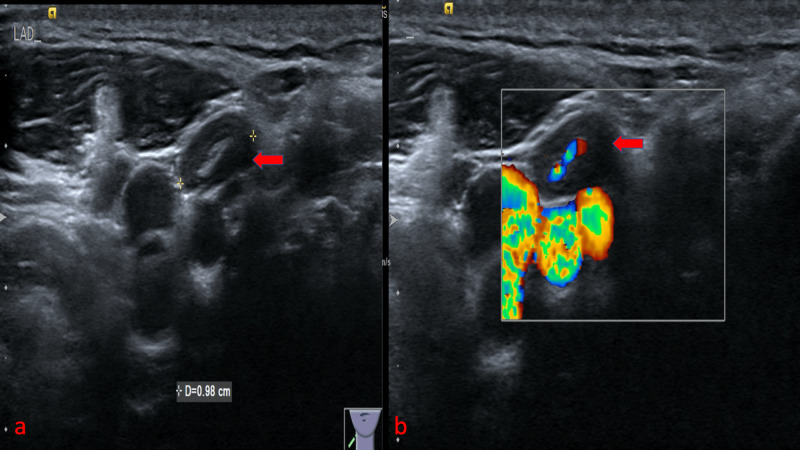
Imaging of normal cervical lymph node: (a) BMUS shows a lymph node with central fatty hilum (red arrow), (b) CDUS shows the hilar flow (red arrow) BMUS: B-mode ultrasound; CDUS: color doppler ultrasound

The presence of ill-defined margins was significantly higher in the malignant nodes than the benign nodes with a p-value <0.001. BMUS and CDUS of the normal lymph node showed central fatty hilum and hilar flow pattern (Figure 4).

**Figure 5:**
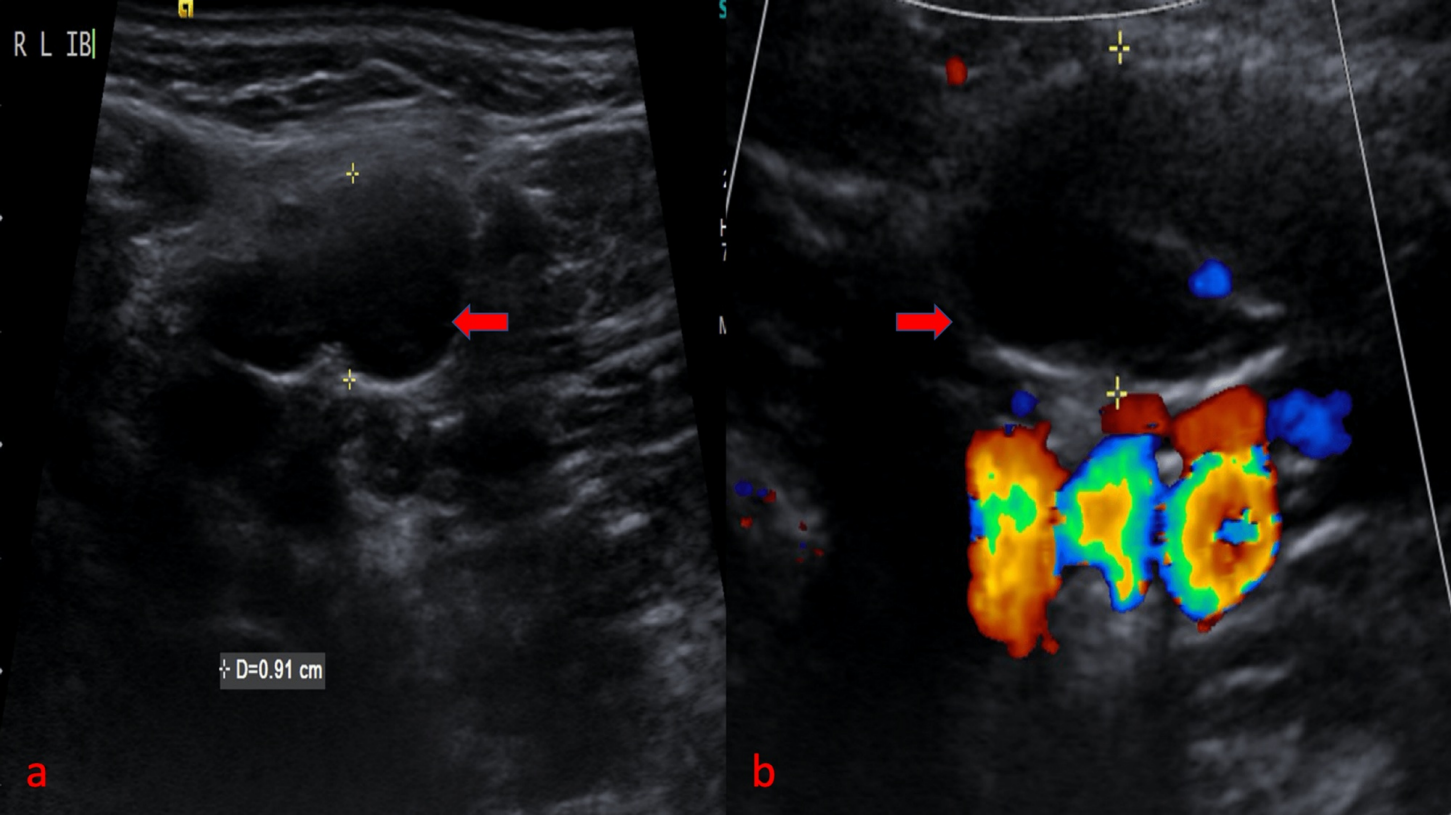
Imaging of reactive cervical lymph node: (a) BMUS shows an enlarged node with compressed hilum (red arrow), (b) CDUS shows hilar flow (red arrow) BMUS: B-mode ultrasound; CDUS: color doppler ultrasound

Similarly, reactive cervical lymph nodes (>5mm in the maximum horizontal axis) showed compressed echogenic hilum (90%) and hilar flow pattern (Figure 5).

**Figure 6:**
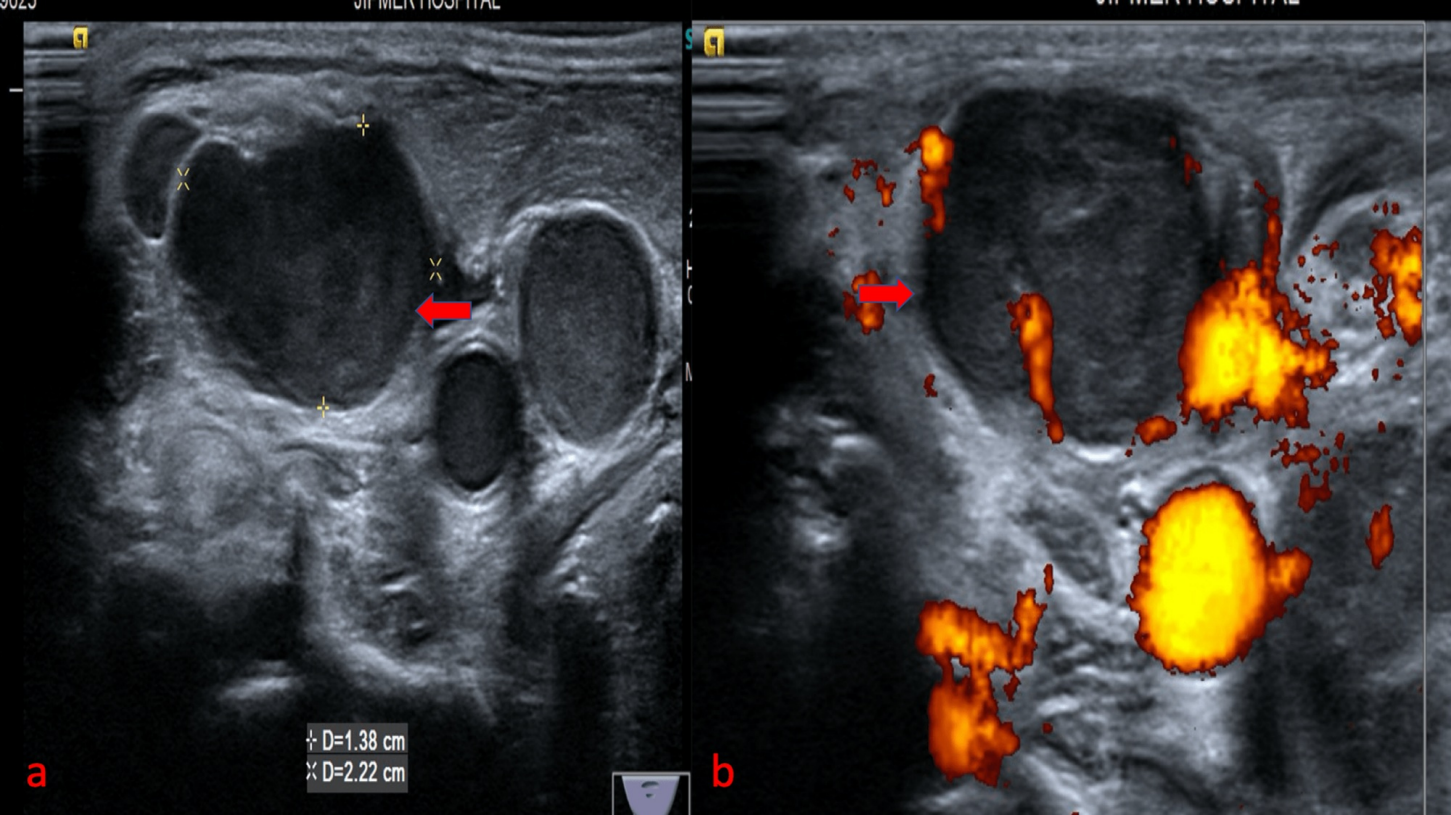
Imaging of tuberculous cervical lymphadenopathy: (a) BMUS shows an enlarged necrotic lymph node adjacent to the common carotid artery (red arrow), (b) CDUS shows the hilar vascularity within the lymph node (red arrow) BMUS: B-mode ultrasound; CDUS: color doppler ultrasound

On imaging the tuberculous lymph nodes, BMUS and CDUS showed enlarged necrotic lymph node with absent or hilar vascularity, respectively (Figure 6).

**Figure 7:**
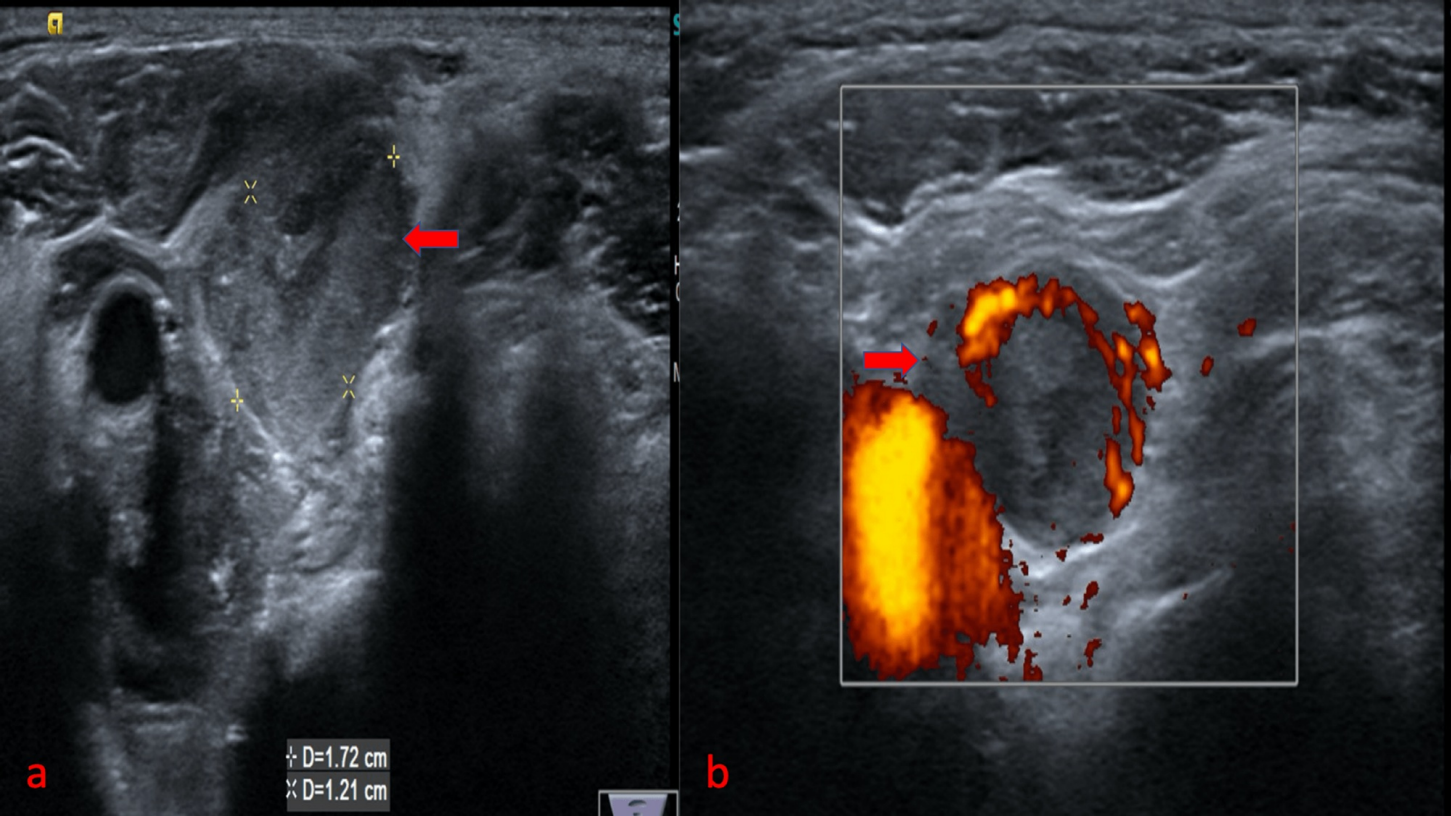
Imaging of metastatic cervical lymph node from Carcinoma hypopharynx: (a) BMUS shows an enlarged heterogeneously hypoechoic node with loss of fatty hilum (red arrow), (b) CDUS shows peripheral vascularity in the node (red arrow) BMUS: B-mode ultrasound; CDUS: color doppler ultrasound

Tuberculous nodes showed a variable internal flow pattern, with 59.2% nodes showing the benign pattern and 40.8% showing a malignant pattern. Among the nodes with peripheral/mixed vascularity, 68.9% of nodes were malignant, and among the nodes with central vascularity, 89.36% of nodes were benign. Malignant cervical lymph nodes showed the loss of echogenic hilum with the peripheral flow in BMUS and CDUS, respectively (Figure 7).

In this study, they found the loss of echogenic hilum to be the most accurate B-mode parameter (79.5%) and the most sensitive of all the parameters (95.4%) in detecting malignant lymph nodes. In comparison, the S/L ratio was found to be the most specific B-mode parameter (74.7%). CDUS had the second-highest sensitivity of all the parameters (94.2%). However, it had poor specificity (53.1%). Summary of the results of BMUS and CDUS are displayed in Table 2.

**Table 2:** Diagnostic value of BMUS and CDUS in malignant cervical lymphadenopathy US: ultrasound; BMUS: B-mode ultrasound; CDUS: color doppler ultrasound; SAD: short-axis diameter; S/L: short-axis to long-axis ratio; ★: area under the curve of 0.732; #: area under the curve of 0.786; Sn: Sensitivity; Sp: Specificity; Acc: Accuracy

S.No	US features		Malignant [N (%)]	Benign [N (%)]	Sn (%)	Sp (%)	Acc (%)	p-value
1	Hilum	Absent	83 (73)	30 (27)	95.4	62	79.5	<0.001
		Present	4 (8)	49 (92)				
2	Borders	Ill-defined	49 (69)	22 (31)	56.3	72.1	63.8	<0.001
		Well-defined	38 (40)	57 (60)				
3	Color doppler	Mixed/Peripheral	82 (69)	37 (31)	94.2	53.1	74.7	<0.001
		Hilar	5 (11)	42 (89)				
4	SAD	≥1.23cm	61 (70)	26 (30)	70.1	67.1	68.7	<0.001^★^
		<1.23cm	26 (23)	53 (67)				
5	S/L	≥0.595	69 (77.5)	20 (22.5)	79.3	74.7	77.1	<0.001^#^
		<0.595	18 (23)	59 (77)				
6	Echogenicity	Hypoechoic	76	69	-	-	-	0.998
		Hyperechoic	11	10	-	-	-	

## Discussion

BMUS and CDUS assessed morphological and vascular features of each lymph node. Palpation has a sensitivity of 73% in determining the nodal characteristics. The US has higher sensitivity (97%) to distinguish benign and malignant cervical lymph nodes. Even though ultrasound is comparatively much more sensitive for this purpose, no single parameter is sufficient for distinguishing benign and malignant lymph nodes. When FNAC is carried out under US guidance, a specificity as high as 93% could be achieved [6-8].

BMUS can help in assessing the location, size, shape, borders, echogenicity and the hilum of the nodes [1]. The cut-off values for the normal size of the cervical nodes vary across the literature [5]. Malignant nodes can be better distinguished from the benign nodes if the SAD cut-offs used are different for each level. The accuracy was 84% in one such study by Alam et al. [9]. When a common cut off of 8mm for all cervical levels was used in the study by Lyshchik et al., the accuracy was 65% [3]. They found a SAD cut-off of 1.23cm to identify a malignant cervical node with sensitivity, specificity and accuracy of 70.1%, 67.1% and 68.7%, respectively. The smallest and the most significant benign nodes in the study were reactive lymph nodes measuring 0.5cm and 5.2cm in SAD, respectively. In contrast, the smallest and the most significant malignant nodes were metastatic adenocarcinomas, measuring 0.5cm and 6cm in SAD, respectively. As many benign reactive and inflammatory nodes may be large, while malignant nodes may be small, the only size cannot differentiate benign from the malignant nodes [5].

Malignant nodes are generally rounded, with an L/S ratio of <2 or an S/L ratio >0.5 [3,5,7]. They found an S/L cut-off of 0.5950 in their study with a sensitivity, specificity and accuracy of 79.3%, 74.7% and 77.1%, respectively. S/L was the most specific B-mode parameter in this study, akin to a study by Lyshchik et al. who found S/L ratio to be the best B-mode parameter with a sensitivity of 75%, specificity of 81% and an accuracy of 79% [3]. In some cases, the shape of a node may be misleading, e.g., rounded nodes with S/L >0.5 may be seen in case of tuberculosis, Kimura’s disease and Rosai-Dorfman disease [10]. A few normal submandibular and parotid group nodes may be rounded. In a study, Ying et al. reported that 95% of the normal level IB nodes are rounded [11]. Hence, the node's shape alone cannot be considered in differentiating the benign and malignant lymph nodes [5,11].

The echogenic hilum of a normal lymph node is due to the interface of multiple sinuses in the medulla. Normal cervical nodes >5mm in the maximum horizontal axis show the echogenic hilum in 90% of cases [5]. Loss of echogenic hilum had an accuracy of 79.5% in identifying a malignant cervical node in this study, comparable to 86% in a study by Alam et al. [9]. However, it had a low specificity of 62%. This was in concordance with the previous studies suggesting that lack of hilum had high sensitivity and low specificity for malignant nodes [3,5].

Among the nodes with ill-defined margins, 69% were malignant, and among the nodes with well-defined margins, 40% were malignant. In studies by Mazaher and Sharifian and Teng et al., ill-defined margins had a statistical significance with malignant nodes, comparable to this study [2,12]. Several studies have shown malignant cervical nodes to have a more well-defined border than the benign nodes [3,4,13]. Unlike in these studies, they found more malignant nodes to have ill-defined borders, probably due to differences in the histopathological types and infiltrative nature of malignant cases in their study population. 

Various studies have shown that vascular flow pattern helps in distinguishing benign and malignant nodes, with a sensitivity of 78 to 96% and a specificity of 76 to 98% [14-17]. Normally, a lymph node shows hilar vascularity. Benign lymph nodes generally tend to have a normal flow pattern. Nearly 96% of the reactive nodes show hilar flow and seldom show peripheral vascularity [1,3]. Malignant lymph nodes, on the other hand, show neo-vascularity. Hilar vascularity is usually disrupted in these nodes. Tumour angiogenesis results in peripheral vascularisation of the nodes. Pre-existing peripheral vessels or vessels in the peri-nodal soft tissues may be contributing to this process. With the progression of the lymph nodal involvement, both the peripheral and the central zones show increased vascularity [1]. A few metastatic nodes may show no detectable internal flow. This may be due to necrosis or due to replacement by keratinized tumor tissue [18]. Even tuberculous nodes showed a variable pattern of internal flow, with 59.2% nodes showing the benign pattern and 40.8% showing a malignant pattern [11]. This study found more tuberculous nodes showing a peripheral/mixed pattern (66.6%). By other studies, the presence of peripheral/mixed vascularity was higher in the malignant nodes as compared to the benign nodes with a p-value <0.001. In a study by Lyshchik et al., 96.5% of nodes with peripheral vascularity were metastatic, and 71.4% of nodes with hilar/absent vascularity were benign [3].

Limitations

Due to the small sample size in various sub-groups of benign and malignant lymph nodes, it was not possible to do subgroup analysis for each histological type. The predictive role of individual BMUS and CDUS parameters were assessed for differentiating the benign and malignant nodes. They did not attempt to do any multivariate analysis or scoring system based on BMUS and CDUS. They did not assess the role of CDUS indices like the resistive index and pulsatility index in the differentiation of cervical lymph nodes.

## Conclusions

Malignant nodes had significantly higher SAD, higher S/L ratio, loss of echogenic hilum, presence of ill-defined margins and peripheral/mixed vascularity compared to benign nodes. The loss of echogenic hilum was the most accurate and sensitive parameter, while the S/L ratio was found to be the most specific BMUS parameter in the detection of malignant nodes. BMUS and CDUS identifies malignant nodes, helps in guiding FNAC/biopsy and can potentially reduce unnecessary biopsies. It also helps in selecting the most appropriate treatment for each patient.
